# Supplementary material for: Determinants of DDX3X sensitivity uncovered using a helicase activity in translation reporter
Source: bioRxiv. 2023 Sep 14:2023.09.14.557805. Preprint. [Version 1] doi: 10.1101/2023.09.14.557805 (PMC10515938; doi:10.1101/2023.09.14.557805)
Supplement: Supplement 1 [file NIHPP2023.09.14.557805v1-supplement-1.pdf]

bioRxiv preprint doi: https://doi.org/10.1101/2023.09.14.557805; this version posted September 14, 2023. The copyright holder for this preprint (which was not certified by peer review) is the author/funder, who has granted bioRxiv a license to display the preprint in perpetuity. It is made available under aCC-BY 4.0 International license.

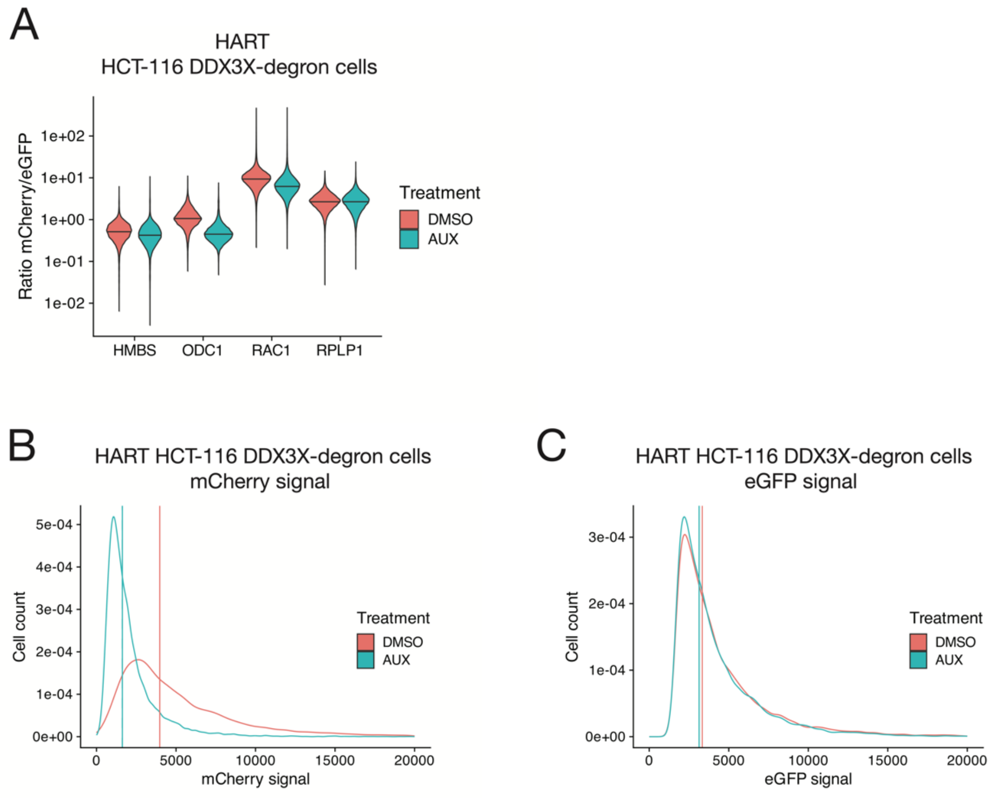

Supplementary Figure 1. (A) Violin plot for HART ratio in HCT116 degron cells analyzed with flow cytometry for the experiment in Figure 1B. HCT116 degron cells were lentivirally transduced with HART constructs with various 5′ UTRs in front of mCherry. After 48 hours from the addition of either DMSO or auxin, which induces degradation of endogenous DDX3X, the fluorescent signal was measured by fluorescent cytometry. The HART ratio (mCherry/eGFP) was calculated for each cell and plotted as a violin plot. **(B)** Flow cytometry data for the mCherry channel for experiment in Figure 1B. The raw data for the 561nm 50mW laser, YG C detector (corresponding to mCherry) for each cell was plotted and the mean was calculated and plotted as a vertical line. **(C)** Flow cytometry data for the eGFP channel for cells in Figure 1B. The raw data for the 488nm 60mW laser, Blue C detector (corresponding to eGFP) for each cell was plotted and the mean was calculated and plotted as a vertical line.

bioRxiv preprint doi: https://doi.org/10.1101/2023.09.14.557805; this version posted September 14, 2023. The copyright holder for this preprint (which was not certified by peer review) is the author/funder, who has granted bioRxiv a license to display the preprint in perpetuity. It is made available under aCC-BY 4.0 International license.

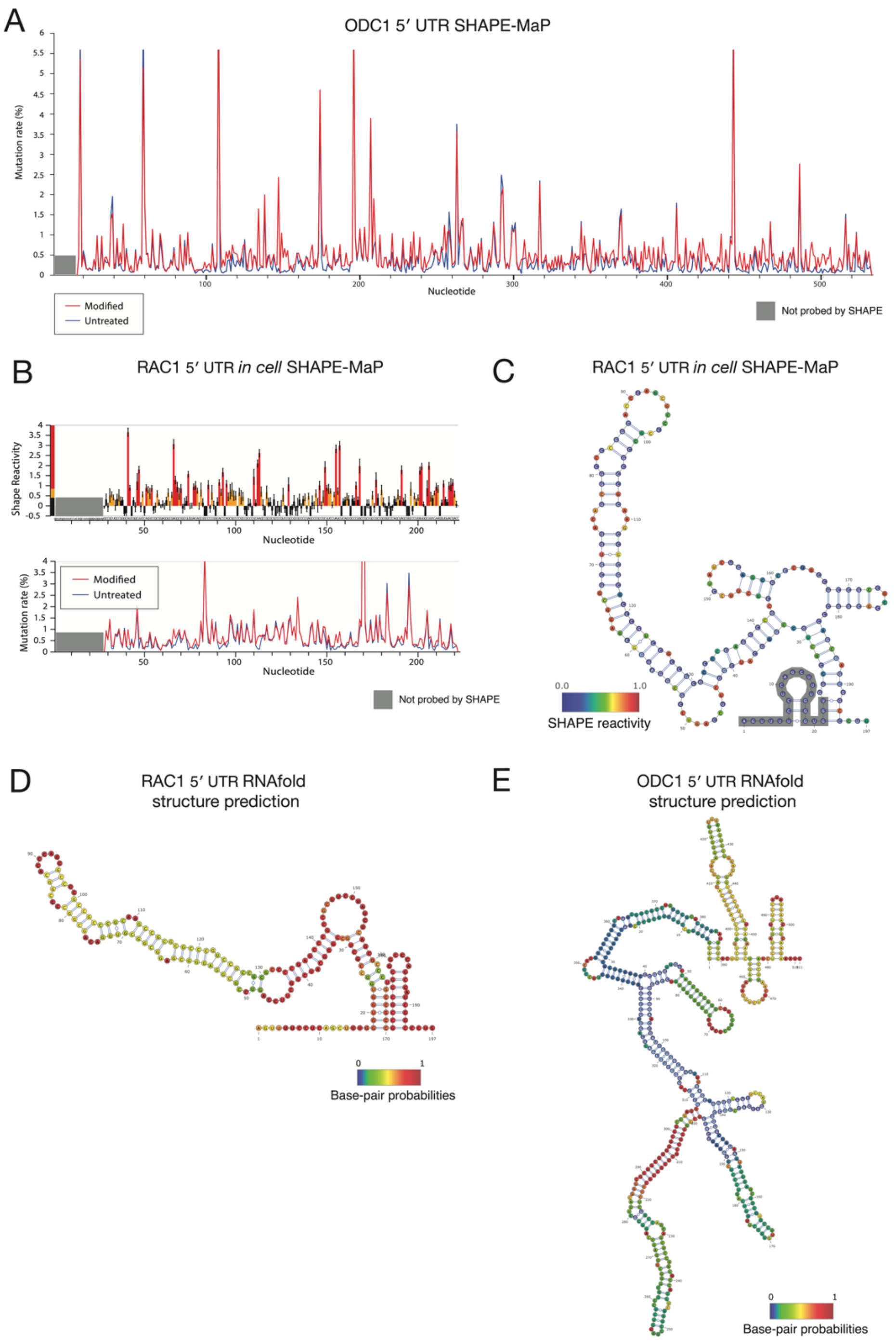

Supplementary Figure 2 (A) SHAPE-MaP reactivity for the 5′ UTR of RAC1 *in vitro* from Figure 2C. *In vitro* transcribed mRNA containing the 5′ UTR of ODC1 and the open reading frame of luciferase was probed with 200 mM NAI or DMSO control for SHAPE-MaP. The RNA was reverse transcribed and sequenced. The SHAPE reactivity was calculated based on the difference in mutation rate. **(B)** SHAPE-MaP reactivity and mutation rate for the 5′ UTR of RAC1 *in cell*. Cells were treated with 300 mM NAI in PBS or control for 20 min before quenching the reaction and extracting the RNA. The RNA was reverse transcribed, sequenced, and analyzed to obtain mutation profiles and SHAPE reactivity with the ShapeMapper tool. **(C)** Diagram of the structure of the RAC1 5′ UTR *in cell*, based on data from Supplementary Figure 2A and computed with ShapeMapper 2.1.3. (Busan & Weeks, 2018) **(D-E)** RNA folding minimum free energy prediction of the structures of the 5′ UTRs of RAC1 (C) and ODC1 (D) using the ViennaRNA Package (Lorenz et al., 2011). The minimum free energy for the RAC1 structure is -97.20 kcal/mol and for the ODC1 is -251.70 kcal/mol.

bioRxiv preprint doi: https://doi.org/10.1101/2023.09.14.557805; this version posted September 14, 2023. The copyright holder for this preprint (which was not certified by peer review) is the author/funder, who has granted bioRxiv a license to display the preprint in perpetuity. It is made available under aCC-BY 4.0 International license.

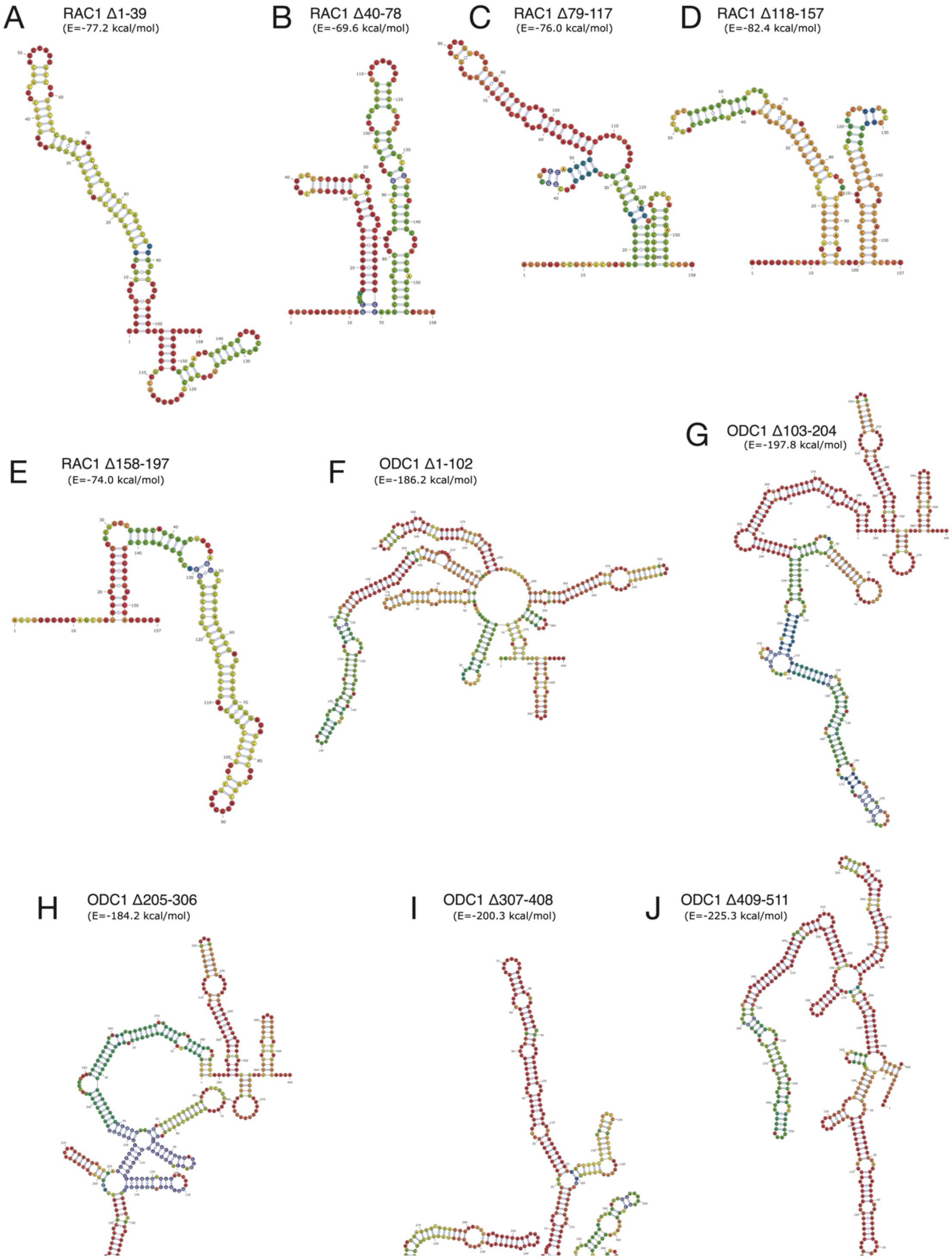

Supplementary Figure 3 (A-E) RNA folding minimum free energy prediction of the structures of the 5′ UTR of RAC1 containing the deletions used in Figure 3 B-C using the ViennaRNA Package (Lorenz et al., 2011). The predicted minimum free energies for the RAC1 deletion constructs are: -77.20 kcal/mol for Δ1-39 -77.20 kcal/mol, -69.60 kcal/mol for Δ40-78, -76.00 kcal/mol for Δ79-117, -82.40 kcal/mol for Δ118-157, -74.00 kcal/mol for Δ158-197. **(F-J)** RNA folding minimum free energy prediction of the structures of the 5′ UTR of ODC1 containing the deletions used in Figure 3 B-C using the ViennaRNA Package (Lorenz et al., 2011). The predicted minimum free energies for the ODC1 deletion constructs are: -186.20 kcal/mol for Δ1-102, -197.80 kcal/mol for Δ103-204, -184.20 kcal/mol for Δ205-306, -200.30 kcal/mol for Δ307-408, -225.30 kcal/mol for Δ409-511.

bioRxiv preprint doi: https://doi.org/10.1101/2023.09.14.557805; this version posted September 14, 2023. The copyright holder for this preprint (which was not certified by peer review) is the author/funder, who has granted bioRxiv a license to display the preprint in perpetuity. It is made available under aCC-BY 4.0 International license.

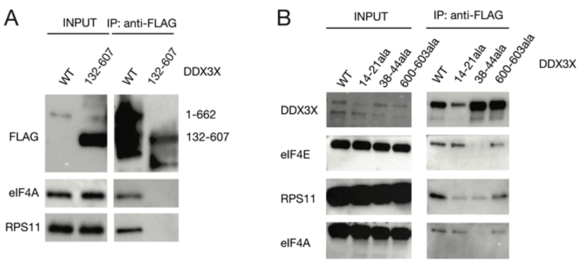

Supplementary Figure 4 (A) DDX3X full length and 132-607 immunoprecipitation. HEK 293T cells were lentivirally transduced with FLAG tagged DDX3X full length or DDX3X 132-607, which represents its functional helicase core. Immunoprecipitation was conducted for FLAG and run on western blot, staining for ribosome-related proteins and controls. **(B)** Immunoprecipitation of DDX3X mutants. HEK 293T cells were lentivirally transduced with FLAG tagged DDX3X WT or several mutants, including the helicase defective mutant R534H and three N- and C-termini mutations in sites conserved across the DDX3X/Ded1 subfamily. Immunoprecipitation was conducted for FLAG and run on western blot, staining for ribosome-related proteins and controls.

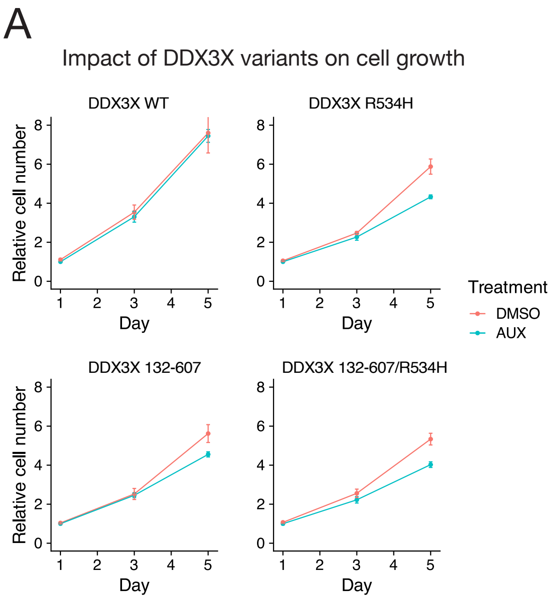

Supplementary Figure 5 (A) Cell growth curves for DDX3X variants. HCT116 degron cells where lentivirally transduced with exogenous DDX3X WT and mutants. Auxin was added to induce loss of endogenous DDX3X and cell number was measured over time with CellTiter Glo.
